# Synchronous citizen science with dogs

**DOI:** 10.1007/s10071-024-01882-6

**Published:** 2024-07-06

**Authors:** Madeline H. Pelgrim, Zachary Tidd, Molly Byrne, Angie M. Johnston, Daphna Buchsbaum

**Affiliations:** 1 Department of Cognitive, Linguistic, & Psychological Sciences, Brown University, 190 Thayer St. , Providence, RI 02912 USA; 2 Department of Psychology and Neuroscience, Boston College, Chestnut Hill, MA USA

**Keywords:** Canine Cognition, Citizen Science, Domestic Dog, Social Cognition, Decision Making

## Abstract

Citizen science approaches have grown in popularity over the years, partly due to their ability to reach a wider audience and produce more generalizable samples. In dogs, these studies, though, have been limited in their controls over materials or experimental protocols, with guardians typically reporting results without researcher supervision. Over two studies, we explored and validated a synchronous citizen science approach. We had dog guardians act as experimenters while being supervised by a researcher over Zoom. In study 1, we demonstrated that synchronous citizen science produced equivalent levels of performance to in-lab designs in a choice task. Consistent with past in-lab research, dogs selected a treat (vs. an empty plate) in a two-alternative forced-choice task. In study 2, we showed that Zoom methods are also appropriate for studies utilizing looking time measures. We explored dogs’ looking behaviors when a bag of treats was placed in an unreachable location, and dogs’ guardians were either attentive or inattentive while dogs attempted to retrieve the treats. Consistent with past work, dogs in the attentive condition looked at their guardian for longer periods and had a shorter latency to first look than dogs in the inattentive condition. Overall, we have demonstrated that synchronous citizen science studies with dogs are feasible and produce valid results consistent with those found in a typical lab setting.

**Supplementary Information:**

The online version contains supplementary material available at 10.1007/s10071-024-01882-6.

Citizen science, or engaging with members of the public to participate in the scientific process by conducting research or gathering data, has become increasingly popular. Citizen science can increase community engagement and can allow for the collection of more naturalistic data from more generalizable populations, thanks to the lack of geographic restrictions in collecting data (Cohn 2008; Silvertown 2009).

One species that is ideal for leveraging citizen science is dogs (Hecht and Spicer Rice 2015). Dogs have a unique social relationship with humans, living closely with us in or around our homes and serving in various working roles. In companion animals like dogs and cats, citizen science, particularly where guardians (owners and/or those in the household who care for the pet) carry out a study with their own pet, has some significant potential advantages over studies conducted in a laboratory environment, such as providing larger samples and more naturalistic data by collecting the data in the animal’s daily environment (Smith et al. 2021, 2022; Stewart et al. 2015). In addition, the ubiquity of dogs around the world also provides a wealth of opportunities for humans to observe and interact with dogs and to explore learning in non-human animals (ManyDogs Project et al. 2023).

Despite this, most cognitive studies with pet dogs have been conducted in a laboratory context. One reason for this may be that laboratory-based studies are conducted in a more controlled environment and carried out by trained research staff, allowing for greater precision and consistency across individual dogs. In contrast, studies conducted by dog guardians can be highly variable and lacking in data controls. One possible solution to this limitation is to conduct synchronous citizen science research, where a trained researcher instructs and supervises the guardian to act as an experimenter (see Fukimoto et al. 2023; for a similar approach in another companion species, cats). In the present studies, we validate the feasibility and accuracy of using a remote supervised approach to guide dog guardians to act as experimenters (Byrne et al. 2023; Pelgrim et al. 2023). In the following sections, we first provide some additional background and context for both laboratory-based and citizen science research. We then present results from two initial validation studies using this approach. We have chosen two tasks, an object tracking task and an unsolvable task, where prior work has established strong expectations for dogs’ performance and are conceptually replicating these tasks in the home environment with a virtual experimenter. Finally, we discuss the relative advantages and limitations of a synchronous citizen science approach and provide suggestions for future researchers.

## Background

For a typical study conducted in a laboratory context, dog guardians^1^ bring their dogs into the lab, and after some acclimation period, dogs participate in studies that usually take the form of treat-finding games. In these games, dogs may solve a physical problem (like a puzzle box) or receive social information from a human informant (see Bensky et al., 2013 for a review). Dogs’ behaviors in response to the problems they are presented with are recorded and analyzed. These behaviors range widely, including choices (where the task is typically a forced choice between a series of alternatives) and looking behaviors (where time spent looking at or away from target objects is recorded).

From this work, we know that dogs are skilled social learners and are sensitive to various characteristics of human informants, such as their accuracy (Pelgrim et al. 2021) and prosociality (Silver et al. 2020). For instance, dogs have an exceptional ability to respond to human social-communicative gestures, particularly when compared to wolves and our closest genetic relatives, the great apes (Bräuer et al. 2006; Hare et al. 2002). Dogs are also sensitive to human gaze patterns. For instance, dogs can take on the visual perspective of a person, using this ability to selectively trust someone who saw food being hidden vs. someone who didn’t (Catala et al. 2017; Maginnity and Grace 2014). Dogs can also use the human gaze as a cue for the person’s attentional state. Dogs beg more from experimenters looking at or facing them than experimenters who do not (Bräuer 2014; Gácsi et al. 2004).

In addition, dogs are less likely to follow the commands of an experimenter facing away from them (Yamamoto et al. 2011). In-lab research has also explored canine cognition in a developmental context. From puppyhood, dogs can learn how to solve novel problems by observing conspecifics (other dogs) and humans (Bray et al. 2021; Fugazza et al. 2018). Dogs tend to prioritize independent problem solving; however, when faced with impossible tasks, dogs give up and look back to human partners, something that has been suggested as a kind of social problem solving strategy (Johnston et al. 2021; Miklósi et al. 2003; Passalacqua et al. 2011). When seeking help from human partners, dogs are selective in who they look back to. In an ability they share with human children, dogs preferentially look to attentive (vs. inattentive) people when a previously solvable task becomes unsolvable (Marshall-Pescini et al. 2013).

Overall, research in the lab has significantly advanced our understanding of canine cognition and learning as a whole. In recent years, however, in part due to the COVID-19 pandemic, researchers have begun exploring new methods for studying canine cognition in alternative contexts. Further, a large part of the motivation behind studying canine cognition is that, unlike other animal species commonly explored in research, dogs typically live in homes with people. Introducing dogs to the lab environment can be stressful, and for dogs with anxiety or aggression, participating in lab-based cognitive studies is often not possible. Exploring dogs’ cognitive abilities in an environment that they are comfortable in may help to improve task performance and increase the kinds of dogs that can participate. Further, by removing travel requirements, dogs who live far away from canine research groups or whose guardians cannot travel can be included in studies.

Citizen science can help to provide a more representative sample, improving the generalizability of scientific results. These approaches allow researchers to gather larger and more diverse samples than they could individually in person. Citizen science also increases the opportunity for community members to engage with the scientific process. In previous citizen science with companion animals, researchers have typically used two variants (Hecht and Spicer Rice 2015). In the first, researchers send details of the study to the animal’s guardian. The guardian then conducted the experiment and input the data of interest directly in response to form questions (Stewart et al. 2015). While this approach was easy for guardians to use and allowed more people to participate with their dogs, it was limited in the kinds of behaviors that could be recorded. For example, future researchers interested in using this approach could not currently use complex measures that are challenging for guardians to note live while they are participating. Measures like looking times or other more nuanced behaviors cannot be used with this method. Further, with these approaches, subsequent re-coding or verification of the owner’s experimental procedure and inputted results is impossible because the only data experimenters have access to is input by guardians. Because of this limitation, some citizen science projects have used the second major variant, asking guardians to submit video data of the experiment to be coded by a researcher (Hecht and Spicer Rice 2015; Smith et al. 2021, 2022). This has the advantage of providing strict quality control on the data used. However, when errors are detected, the data has to simply be excluded.

Relative to in-lab research, citizen science is limited by a lack of controls. By nature, each participant is being tested in spaces with different sizes and spatial layouts, lighting conditions, and other potentially differing spatial and perceptual features. This may impact performance and overall data quality (Smith et al. 2022). There are also limited controls when guardians act as active experimenters (e.g., are actively presenting stimuli as seen in Stewart et al. (2015) vs. recording responses to stable stimuli like in Smith et al. (2021,2022). Guardians typically receive written and/or video instructions, which can be misunderstood. A further limitation of traditional asynchronous citizen science, particularly when considering guardians as active experimenters, is an inability to remedy errors. In contrast to in-lab research, where mistakes can be fixed and trials can be repeated, guardian experimenters participating in asynchronous research likely do not know or recognize if an error is made.

Synchronous citizen provides many of the advantages of traditional citizen science approaches (i.e., naturalistic data in an ecologically valid environment, the inclusion of a broader population, etc.); however, it also provides the opportunity for advanced and remedial instructions. As mentioned above, this method has previously been successfully conducted in another companion species, cats (Fukimoto et al. 2023). In their study, Fukimoto et al. (2023) explored feeding and playing behaviors in cats, coding for behaviors like meowing and gazing while minimizing disruptions to the cats’ routines. Synchronous citizen science research may increase confidence in the accuracy of the data, allowing for individual trials to be repeated or excluded as appropriate. Further, having synchronous supervised testing can help researchers avoid having to exclude data due to common problems like misplaced camera angles. Finally, a synchronous approach may allow for more complex experimental procedures, as continued instructions can be provided during the session.

In the present studies, we are using a remote supervised approach, specifically using the video-conference software Zoom to guide dog guardians to act as experimenters (Byrne et al. 2023; Pelgrim et al. 2023). We have chosen two well-established tasks in the dog literature that allow us to validate the accuracy of synchronous citizen science and compare the features of the task, such as exclusion rates and timing. In study 1, we evaluated dogs’ performance on an object tracking task, requiring them to make a two-alternative forced choice task between a plate with a treat placed on it and an empty plate. In study 2, we explored dogs’ social behaviors in a problem solving task, specifically their tendency to look back to their guardians on a naturalistic variation of an impossible task (a common paradigm where dogs are presented with an engaging problem that through experimental manipulations they cannot solve independently). These tasks (an object-choice task and a looking-time-based task) are representative of typical canine behavior studies conducted in a laboratory setting. Having disparate tasks is reassuring for validity as it suggests reliability on tasks that differ in format, structure, and dependent variables. By presenting them together, we aim to demonstrate that the synchronous citizen science approach is a valid and accurate method for data collection, enabling researchers with a variety of questions to gather generalizable data from diverse populations in a timely fashion.

## Study 1: object choice task

In study one, we aimed to explore if dogs’ performance on an object-search task at home was comparable to in-lab. We presented dogs with a two-alternative forced-choice task between a treat and an empty plate, similar to previous in-lab tasks (Espinosa et al. 2021). We chose this task because dogs are generally successful at tracking the desired object on this task when it is performed in the lab (~ 83% correct, Espinosa et al. 2021). Dog guardians were trained to act as experimenters and presented their dogs with 12 test trials. We predicted that dogs would perform similarly to in-lab, meaning that they would choose the plate with the treat more than the empty plate. However, it was also possible that dogs would perform more poorly on the task when it is presented at home by their guardians, potentially due to the increased distractions in the home environment or due to increased experimenter variability. Finally, it was possible that dogs might perform better in their home environment, as they may have reduced levels of stress and anxiety when completing the task in a familiar context (vs. in the lab). Unlike in labs, where there are typically trained researchers acting as the experimenter and handling the dog, some dog households only had one person available to act as an experimenter. It was possible that this variability would impact our overall results; for example, dogs without handlers were much worse at the task than dogs with a second person present to handle them. Alternatively, it was possible there were no differences in task performance between these two groups, meaning that synchronous citizen science approaches with dogs can include single-person households.

## Methods

### Participants

Participants were 118 pet dogs (59 Female, Mean Age = 62.47 Months). Participants came from 3 countries and 9 states in the U.S. and the District of Columbia. See Supplementary Materials for breed distribution. An additional 18 dogs were excluded from the study due to (1) guardian-experimenter error resulting in the dog eating the treat after choosing the empty plate (10 dogs were excluded for this reason) or resulting in the dog being unable to eat the treat after choosing it as the plate was taken away incorrectly (1 dog was excluded for this reason), (2) dog failed to complete all warm up and test trials (6 dogs were excluded for this reason), (3) video-experimenter error resulted in a skipped trial (1 dog was excluded for this reason). This exclusion rate (around 13%) is only slightly higher than previous in-lab studies (9% seen in Espinosa et al. 2021).

### Testing set up

**Fig. 1:**
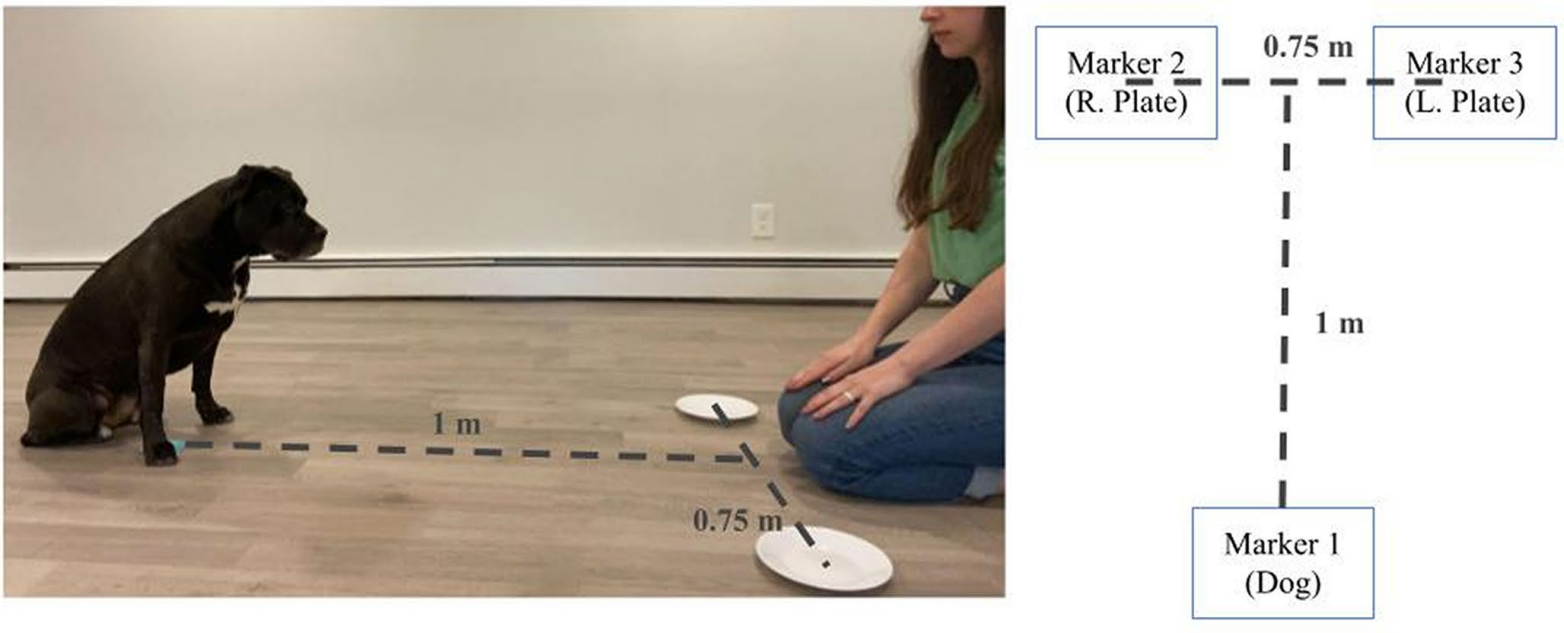
Experimental Setup for Study 1. *Note* **Left** - Example photo of the setup with a dog and guardian. **Right** - Schematic of the setup from a top-down perspective. Dog guardians placed the 3 markers (1 for the dog to stand on, one for each of the 2 plates to be placed on) in advance. The Zoom-enabled device was placed so that both the plates and the dogs’ faces were all visible.

The study was conducted in an open room in the guardian’s home and was completed in a single Zoom session lasting approximately 15–20 min. In advance of the session, dog guardians were sent an instructional document listing the materials required for the study and some example images of potential camera angles. This also provided guidance on selecting an appropriate space for the study, namely choosing a room that the dog is comfortable in and that won’t be disturbed. Guardians were also informed of the total number of treats their dog was likely to receive and instructed to avoid giving them a full meal for a few hours prior to the session. Once connected on Zoom, the virtual experimenter confirmed that the room selected was not likely to be disturbed by other pets or people. The instruction sheet and script used during our sessions are available in our supplementary materials. Then, dog guardians were instructed to mark their floor with three pieces of tape or sticky notes. These three marks formed the endpoints of a T-shape (see Fig. 1, Right). This mimicked the procedure used in a lab environment and helped guardians to be consistent in their placement and setup on each trial. Guardians sat at the center of the top of the T-shape. Dogs waited at the base of the T shape on top of the bottom marker, approximately 1 m away from their guardian. Markers for the plates were located approximately 0.75 m apart from each other. Filming was done through Zoom on a guardian’s laptop, tablet, or device that can record clearly.

### Procedure

Dog choices were defined throughout our study as making physical contact with either the food item or the plate the food item was on. Choices were recorded by the experimenter on Zoom. In the event that a dog failed to make a choice for > 30 s, the trial was repeated (this occurred on 6 out of 1416 trials). Dogs waited while the guardian placed down plates with treats as instructed by a researcher over Zoom. Treats were chosen by the dog guardian for each dog and were cut into bite-sized pieces. When a second person was available, dogs were held in their waiting position by this second person. Otherwise, guardians were instructed to have their dog stay at the waiting position using the command their dog would respond to best. We explicitly compared task performance between these two groups (see Results). Trials began with the plates out of reach of the dog. Dog guardians were instructed by the researcher to set up the plates before placing them on the markers by placing a treat onto one plate. They would then place the plate (in the warm-up) or both the plate with the treat and a second empty plate (test trials) as instructed by the researcher over Zoom. After the guardian completed the placement of plates, they released their dog to make a choice.

To familiarize dogs and guardians with the procedure and ensure the dog was motivated to participate in the task, we first conducted warm-up trials. During warm-up trials, dogs were shown a single plate with a treat. Dog guardians would place the treat onto the plate out of reach of their dog (typically behind their backs) and then place the pre-loaded plate directly in front of where the guardian was sitting. Once the plate was on the ground, the guardian verbally released their dog from the waiting position to eat the treat off of the plate. We repeated this procedure for a minimum of 3 trials, or until both guardians and dogs were comfortable (maximum of 6 trials observed in our sample).

After completing warm-up trials, the guardian presented two plates placed just in front of their knees. As in the warm-up trials, the guardian began by putting a treat onto one of the plates. The guardian then placed the plates, one of which had a treat on it and the other, which was empty, sequentially, as directed by an experimenter over Zoom. The order of placement (treat or empty plate placed first) and the side the treat was on was counterbalanced. After placing both plates onto the ground, the guardian verbally released their dog. If the dog chose the plate with the treat on it, they were allowed to eat the treat. If the dog chose the empty plate, the plate with the treat was removed, and they did not receive any treats on that trial.

### Data coding & analysis

As mentioned above, dogs’ choices were classified as making physical contact with either the plate or the item on the plate. The experimenter recorded the initial choices over Zoom. 25% of videos (*n* = 30 dogs) were re-coded for dogs’ choice by a naive coder. Interrater reliability was very high (358 / 360 trials, 99.44%). In the event of any disagreement between the original Zoom experimenter and the re-coder (occurred on 2 / 360), the trials were re-coded by the first author (MHP), and these codings are reported below.

All statistical analyses were conducted in R statistical software version 4.1.2 (R Core Team 2021). We were first interested if the dogs chose the treat (vs. the empty plate) significantly more than chance levels using a t-test and how this compared to past in-lab work. Next, we were interested in factors that could impact dogs’ choice of the treat, using a linear regression (R package ‘stats’, R Core Team 2021). Our two predictors of interest were whether the dog had a handler (a second person available to hold the dog) and the number of warm-up trials the dog completed. Finally, we wanted to explore if dogs improved over the course of trials. We conducted a generalized linear mixed model with a binomial distribution (treating choice of the treat as correct or 1) using the R package ‘lme4’ (Bates et al. 2015). To control for repeated measures, we included subject identity as a random effect.

## Results

Dogs and guardians were both generally successful at warm-up trials. The majority (*n* = 92 / 118 dogs) completed the warm-up in our predetermined minimum of 3 trials. Further, the maximum number of warm-up trials required in our sample was 6 trials (*n* = 2 / 118 dogs). During our object choice task, on average, dogs chose the treat M = 10.06 / 12 trials or 84%, S.E. = 0.19. This is consistent with past findings from in-lab work, where dogs chose the treat (over the empty plate) on 8.3 / 10 trials (83%) (Espinosa et al. 2021). A one-sample t-test showed that dogs chose the treat plate significantly more than chance levels (chance being 6 / 12 trials or 50%), *t*(117) = 20.87, *p* < .001.

As mentioned above, it was possible that having a handler could impact dogs’ choice of the treat. It was also possible that dogs who had more practice with the behaviors required (through more warm-up trials) may select the treat more often. Alternatively, dogs with increased warm-up trials may have had more difficulty understanding the task overall and could perform worse on average than dogs who required fewer trials. We conducted a linear regression to explore if the dog having a second guardian to handle them (vs. waiting alone) or the number of warm-up trials they completed had an impact on the total number of times they chose the treat. Having both a handler and an experimenter present did not affect dogs’ choice behavior (35 did not have handlers, 83 had handlers), *t*(115) = − 0.48, *p* = .63. We also found no significant effect of the number of warm-up trials dogs completed (M = 3.28, S.E. = 0.06), *t*(115) = -1.30, *p* = .20.

Dogs were also equally successful across trials. We conducted a generalized mixed-effects model exploring dogs’ choices of the treat vs. empty location as predicted by the trial number, with a random intercept for each participant. Using a mixed-effects model, we found that trial number marginally predicted dogs’ choices, *B* = 0.15, *S.E.* = 0.079, *z =* 1.86, *p* = .06. There was also little variance between dogs (SD = 1.25). Dogs are trending towards improvement over the course of trials, choosing the treatment slightly more on later trials (i.e., M_Trial 9_ = 0.88, SD_Trial 9_ = 0.32) than on earlier trials (M_Trial 3_ = 0.78, SD_Trial 3_ = 0.42). However, dogs were very successful at choosing the treat from trial 1 (M_Trial 1_ = 0.86, SD_Trial 1_ = 0.34). This is consistent with in-lab data that also does not find evidence of significant learning across the task (Espinosa et al. 2021).

## Discussion

Overall, our findings were consistent with in-lab research. More specifically, dogs tested by their guardians in their homes were equally successful as dogs in the lab on a simple object search task presented as a two-alternative forced choice. This also suggests that guardians can easily be trained to act as reliable experimenters, at least on simple tasks. Guardians and dogs did not require extensive practice beyond that typically provided in the lab prior to testing. For the majority of dogs, three trials (our minimum, consistent with in-lab work with a trained researcher, Espinosa et al. 2021) were sufficient to have the dog engaged and the guardian comfortable performing the tasks. Further, we found no evidence that having a second person present in addition to the guardian-experimenter, to help position the dog and facilitate their waiting, as is typical in a lab environment, made any impact on their success at locating the treat. Put another way, dogs in our sample did equally well when they had a second guardian present to hold them at their start line as when they waited independently. It is possible that dogs who did have a second guardian present were dogs whom the guardian believed would have struggled to wait independently, and all dogs who waited independently had strong stay/wait commands. What we can conclude from these results is that future remote research can be conducted in single-person households or when only one person is available to conduct the session.

Compared to in-lab sessions, virtual participation requires less time from dog guardians because it eliminates the time required to travel to and from the laboratory. There is also no need to give the dog time to acclimate to the testing space, as sessions are conducted in a familiar room. Our sessions, including instructions and camera angle setup, took on average less than 20 min. Many dog guardians reported that they were participating on their lunch breaks, while their child was napping, or during brief times away from other responsibilities. This approach also allowed people who couldn’t participate in the lab to still participate online, helping to include dogs who live far from our testing center or whose dogs were particularly anxious or otherwise uncomfortable in new spaces.

### Study 2: dogs’ looking back behavior

In Study 2, we aimed to explore if dogs’ looking back behaviors on an impossible task are consistent in homes and in the lab. On a similar task to Marshall-Pescini et al. (2013), we explored if dogs, when presented with an impossible task (specifically a bag of treats placed in an inaccessible location), would look back to their guardian more when their guardian was attentive vs. inattentive. We predicted that dogs would look more at the guardian when the guardian is attentive (vs. inattentive), which is in line with prior findings in the lab environment (Marshall-Pescini et al. 2013). Looking back to an attentive guardian, as seen in Marshall-Pescini et al. (2013) would suggest that dogs are sensitive to their guardian’s attentional states and may be selectively looking back because they are seeking help. Looking back to a guardian can be considered a social tool, seeking help in solving a problem from a social partner (e.g., Miklósi et al. 2003; Passalacqua et al. 2011). Exploring dogs’ looking behaviors in a more naturalistic social setting in response to different social cues from their guardian is particularly well suited for virtual testing. It was possible that dogs would behave differently in a similar setup to Mashall-Pescini et al. (2013) in a more naturalistic setting. This study also allowed us to evaluate the virtual in-home validity of another common paradigm used in lab settings, specifically the recording of looking times. This study was pre-registered^2^.

## Methods

### Participants

Participants were 40 pet dogs (17 males and 18 females, 5 dogs whose guardians did not provide their demographic data, average age 5 years, further demographic data included in supplementary materials). An additional 11 dogs were excluded from the study based on criteria identified in our pre-registration^1^. Specifically, dogs were excluded if (1) Dog was not treat oriented (i.e., they did not eat all of the warm-up treats across all three trials – four dogs were excluded for this reason), (2) Dog successfully pulled out the bag of treats under the furniture (two dogs were excluded for this reason), (3) citizen scientist did not follow directions (one dog), and (4) If the dog remained out of frame for more than half of a given trial (15 s) that trial was excluded, if this persisted across all 3 trials the dog was excluded (four dogs).

In addition, 38 total trials were excluded based on pre-registered criteria. The majority of excluded trials were due to citizen scientist error (i.e. talking during trials, not following directions; 16 trials). In addition, trials were excluded if either the citizen scientist or the dog were not visible on the video for more than half of the test trial (15 s; 10 trials). Trials where distractions in the environment captured the dog’s attention for more than three seconds were excluded (6 trials). If the dog did not eat the warm-up treat, the trial was excluded (4 trials). If the dog solved the impossible task by retrieving the treats on one at trial, the trial was excluded (2 trials).

### Testing set up

The study was done in a spacious room in the living space of the guardian. We used the area under a couch or other piece of furniture as an unreachable area. The dogs could not reach the bag of treats that was placed there without the assistance of their guardian. This is one difference between previous in-lab work, as the size and shape of the bag varied from dog to dog. All bags were shown to the experimenter before filming for approval. As in Study 1, filming was done through Zoom on a guardian’s laptop, tablet, or device that can record clearly. To set up the study in the best way to limit low-quality video recordings, guardians were asked to place laptops or cameras in areas that could capture the dog, guardian, and furniture in the frame. No other people or dogs were allowed to interfere with the experiment, and if they appeared, the trial was excluded as a distraction. We found that the study itself was quick to conduct with dog guardians. On average sessions took less than 15 total minutes (including time for camera angle setup and instructions). The complete script used with dog guardians is available in our supplementary materials.

### Procedure

The study began with warm-up trials to get the dogs focused on the goal of getting treats. Dog guardians were first instructed by a researcher on how to conduct the study over Zoom. Guardians then brought in a plastic bag filled with treats and showed this bag to their dog. It was then placed under the furniture in the unreachable location while the dog was watching. The guardian then gathered two treats from the bag under the couch and placed both treats on the floor in front of the furniture. One treat was placed on the right side of the furniture, and one on the left. After the dog ate both treats, the testing trials, or the Still Phase, began.

**Fig. 2:**
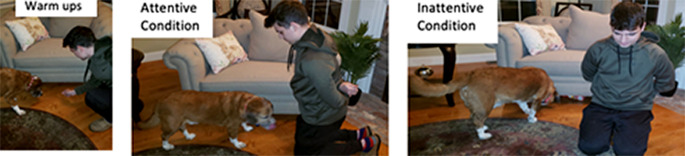
Experimental Setup for Study 2. *Note* Dogs first received warm-up trials (Left). Dogs then experienced either the Attentive (Middle) or Inattentive (Right) condition

During the Still Phase, there were two different conditions: the attentive condition, in which the guardian watched their dog, and the inattentive condition, in which the guardian positioned their body away from the unreachable location and looked down (Fig. 2). 20 subjects were randomly assigned to each group (attentive or inattentive). In the attentive condition, the guardian sat down and watched their dog for 30 s while the experimenter on Zoom kept track of time. Guardians were instructed to keep their hands behind their backs and watch their dogs passively. If their dog made eye contact with them, they were asked to nod and smile back (as in Marshall-Pescini et al. 2013). In contrast, in the inattentive condition, the guardian still sat with their hands behind their back; however, they sat facing in the opposite direction (facing away from the furniture/unreachable location). They were told to look down for 30 s and not make eye contact or communicate with their dog. See Fig. 2 for contrast across conditions. In both conditions, after 30 s had elapsed, the video experimenter instructed the guardian to prepare to re-set for the warm-up phase. This procedure (1 warm-up, 1 Still Phase) was repeated a total of three times.

### Data coding & analysis

Videos from sessions were coded for five behaviors. Dependent variables included (1) Looking back, a state event defined as the amount of time the dog spent with eyes and nose oriented at the guardian above the shoulders (2) latency to first look, a state event defined as the time from the start of the trial (when the dog ate the second treat) until the dog first looked back at the guardian (if the dog did not look on a given trial, they were assigned the maximum duration of 30 s), (3) number of gaze alternations, a point behavior inspired by a coded behavior in a study by Nawroth et al. (2016) where gaze alternation is looking at both the guardian and the unreachable location within a two second period, (4) latency of first gaze alternation, a state event defined as the time it took for a dog to first look at both the guardian and the unreachable location within a 2 s time window (if the dog did not gaze alternate on a given trial, they were assigned the maximum duration of 30 s), and (5) attempted solving behaviors, a state event defined as the amount of time the dog’s front paws or nose went under the barrier (e.g., couch). The five behaviors were all coded for the three trials using BORIS (Behavioral observation interactive software; Friard and Gamba 2016). Two coders coded 100% of all five behaviors for all trials. One coder was the second author (ZT) and the other was a coder blind to the hypothesis of the study. Reliability for the behaviors was *r* = .92 for looking back, *r* = .70 for latency to first look, *r* = .37 for gaze alternations, *r* = .38 for latency to first gaze alternation, and *r* = .81 for attempted solving behaviors. Given our low reliability for some measures, we ran all analyses reported below with both sets of coders’ codes and found the same pattern of significant results for both coders. One likely reason for our relatively low observed agreement is the poor video quality caused by grainy images and poor lighting. This limitation will be discussed further in the general discussion. As video conferencing software and web cameras improve, this will be less of an issue for future researchers, but lower image quality is something to be considered. We report the results from the second author’s (ZT) coding here.

All statistical analyses were conducted using R statistical software version 4.0.3 (R Core Team 2021). Predictors of interest were the guardian’s attentional state (attentive or inattentive), trial number, and the interaction between attentional state and trial number. This potential interaction allowed us to explore if dogs learned differentially over time that their guardian wasn’t reacting to their behavior (e.g., if during the attentive condition, dogs stopped looking over time because their guardian was not helping them). Data were analyzed for each of the five dependent variables with linear mixed models (LMM) using the R package ‘lme4’ (Bates et al. 2015). Gaze alternation was fit as a Poisson distribution given that it is a count variable, and all other variables were continuous distributions. Subject identity was included as a random effect to control for repeated measures. In mixed model analyses, we first examined a null model, which included only subject identity. We then compared the null models with full models that included all predictor variables and their interactions. Model comparisons were conducted with likelihood ratio tests.

## Results

Our models for looking back and gaze alternation revealed that subjects’ looking was predicted by condition (LRTs: *Xs*^2^ > 6.37, *p*s < 0.003), such that dogs looked back longer in the attentive condition (*M* = 5.96, *S*.E. = 0.88) than in the inattentive condition (*M* = 1.05, *S.E.* = 0.51) and alternated their gaze more in the attentive condition (*M* = 0.57, *S*.E. = 0.12) than in the inattentive condition (*M* = 0.17, *S*.E. = 0.06). See Fig. 2. No other factors or interactions were significant predictors for looking back or gaze alternation (LRT: *p*s > 0.35). Our models for latency to look back and latency to gaze alternate revealed that subjects’ looking was predicted by condition (LRTs: *Xs*^2^ > 6.36, *p*s < 0.012), such that dogs had a greater latency to look back in the inattentive condition (*M* = 21.90, *S*.E. = 1.79) than in the attentive condition (*M* = 9.73, *S*.E. = 1.19) and a greater latency to gaze alternate in the inattentive condition (*M* = 25.64, *S*.E. = 1.50) than in the attentive condition (*M* = 19.61, *S*.E. = 1.53). No other factors or interactions were significant predictors for latency to look back or latency to gaze alternate (LRT: *p*s > 0.22). Our full model for solve attempts was no better at predicting solve attempts than our null model (*p* = .277).

## Discussion

In line with the results of Marshall-Pescini and colleagues (2013), we found that when dogs’ guardians were attentive, dogs looked at their guardians for longer periods and had a shorter latency until the first look than when their guardians were inattentive. These findings support the idea that dogs use their looking behaviors as a social tool to recruit assistance from their guardians and that they are able to use the guardian’s attentional states to identify when their human partner is available to be a helper. There is a possibility that dogs simply look back at humans because humans are a salient thing in their environment, and this is associated with prior reward history (Lazzaroni et al., 2020). However, if humans were solely a salient thing in the dog’s environment, then the orientation of the human would not impact the dog’s looking behaviors. Overall, replicating the original findings of Marshall-Pescini et al. (2013) in an in-home context is particularly significant, axs this suggests that this kind of social engagement is part of dogs’ daily lives.

### General discussion

In study 1, we found that dogs are successful on an object search task in an in-home context. We successfully validated the synchronous citizen science approach by finding that it was consistent with past work in the lab (Espinosa et al. 2021) on a two-alternative forced-choice task. Dogs chose the treat significantly more than the empty plate and at rates nearly identical to their performance in a lab environment. This demonstrates that typical object search tasks are feasible to conduct over Zoom with dog guardians acting as experimenters. In study 2, we found that when dogs’ guardians were attentive, dogs looked to their guardians sooner and for longer, as compared to when guardians were inattentive. This supports the idea that dogs are sensitive to guardian attentional states and look back to ask for help. This is also consistent with past work (Marshall-Pescini et al. 2013) and shows that looking-time-based studies can also be conducted in a home environment via Zoom. In sum, our results suggest that Zoom based studies can provide accurate data.

While we found comparable data to in-lab studies, there are obviously some limitations. First - relative to in-lab, there is significantly more environmental variation across participants. Synchronous citizen science approaches capture the natural variability across pet dogs’ home environments, but these environments often vary in ways that can’t be controlled (i.e., the size of the room and furniture, and how isolated the room is from other household members or outdoor noise). Second - this approach may not be appropriate for research questions centered on specific objects or items. Both studies presented here did not require specialized apparatuses or specific equipment, making them easy to conduct with materials that dog guardians have in the home already. This may be more naturalistic, as they are objects the dogs will encounter. However, research questions requiring tightly controlled demonstrations or specific stimuli may not be appropriate to explore using this method.

Future researchers interested in implementing synchronous citizen science should consider the inherent methodological challenges that could impact their results. As mentioned in Study 2, depending on the device used by the dog’s guardian to make the recording, Zoom based studies can have low-quality video data due in part to poor lighting conditions, issues with the internet connection, or inherent device limitations. This could result in challenges to researchers who are looking to record very fine-grained or short-lasting behaviors, like the looking times recorded in study 2. Partially as a result of the COVID-19 pandemic, most dog guardians contacted were relatively familiar with accessing Zoom or similar software. However, not all were previously comfortable using computers. Additionally, finding the correct camera angle to get both the guardian and dog in the frame was a challenge that did not exist in the lab.

There were also significant advantages to conducting studies over Zoom. We were not constrained geographically when recruiting, and our participants could live anywhere in the world. Further, we were able to include dogs who are anxious or uncomfortable meeting new people that would not have participated in lab. There were also significant advantages when scheduling dog guardians relative to in-lab work, as there was no travel time or logistics required. Dogs did not have to have any acclimation time to the space, something typically required in lab studies. The inclusion of a more diverse sample of dogs in studies conducted via Zoom may help to increase the generalizability of results.

As previously discussed, past citizen science approaches have had guardians collect data on their dogs asynchronously and submit the data (i.e., Stewart et al. 2015). Relative to our Zoom studies, these asynchronous studies require much less time on the part of the experimenter and may be able to get a more global sample as they are not limited in scheduling by time zones. However, by having studies conducted synchronously, we are able to catch errors and clarify instructions with dog guardians before data is submitted. By having a trained experimenter supervise the recording and collection of the data, we can be confident in our results and end up with video data that can be re-coded for reliability after the session. Further, any data where errors did occur was easily excluded and avoided influencing our results.

In sum, over two studies, we have shown that collecting data via Zoom with guardians acting as experimenters provides accurate data, with results highly comparable to those collected in person in the lab. While this method may not be appropriate for all future research questions, it can help researchers acquire larger and more diverse data samples while maintaining a high quality of data. This can improve the generalizability of results in canine science and improve community engagement.

## Electronic supplementary material

Below is the link to the electronic supplementary material.

Supplementary Material 1

## Data Availability

Data for study 1 and study 2 can be found on our project page on the Open Science Framework: https://osf.io/ewycx/?view_only=2ef036a414fe45feb99b555133e6d49e.

## Footnotes

1 Dog guardians refer to dog owners and/or those in the household who care for the dog.

2 Link to the pre-registration for Study 2: https://aspredicted.org/blind.php?x=4gn2kn.
