# Supplementary material for: Synchronous citizen science with dogs
Source: Anim Cogn. 2024 Jul 6;27(1):46. doi: 10.1007/s10071-024-01882-6 (PMC11226520; doi:10.1007/s10071-024-01882-6)
Supplement: Supplementary file 1 — Supplementary Material 1 [file 10071_2024_1882_MOESM1_ESM.docx]

| Breed | Count of Dogs |
| --- | --- |
| Australian Labradoodle | 1 |
| Bichon Frisé | 1 |
| Boston Terrier | 1 |
| Cavalier King Charles Spaniel | 2 |
| Chesapeake Bay Retriever | 1 |
| Cockapoo | 1 |
| Dalmatian | 1 |
| English Cocker Spaniel | 1 |
| English Setter | 1 |
| English Shepherd | 2 |
| English Springer Spaniel | 1 |
| French Bulldog | 2 |
| German Shepherd Dog | 2 |
| Golden Retriever | 3 |
| Goldendoodle | 2 |
| Goldendoodle (miniature) | 1 |
| Havanese | 1 |
| Husky Shephard | 1 |
| Karelian Bear Dog | 1 |
| Labradoodle | 1 |
| Labradoodle (miniature) | 1 |
| Labrador Retriever | 8 |
| llewellin setter | 1 |
| Manchester Terrier | 1 |
| Minature American Shepherd | 1 |
| Miniature Pinscher | 1 |
| Mixed Breed | 57 |
| Newfoundland | 1 |
| Norwich Terrier | 1 |
| Parson Russell Terrier | 1 |
| Pembroke Welsh Corgi | 3 |
| Poodle (miniature) | 1 |
| Poodle (standard) | 2 |
| Portuguese Water Dog | 3 |
| Pug | 1 |
| Samoyed | 1 |
| Schnauzer (miniature) | 3 |
| Smooth Collie | 1 |
| Staffordshire Bull Terrier | 1 |
| Vizsla | 1 |
| Weimaraner | 1 |
| **Grand Total** | **118** |

Table 1. Breed summary for all participants in Study 1.

**Supplementary Materials**

| Order Tested | Participant ID | Condition | Sex | Breed | Age at time of test |
| --- | --- | --- | --- | --- | --- |
| 1 | 3154 | Attentive | Female | Golden Retriever | 2 |
| 2 | 3115 | Inattentive | Female | Mixed | 9 |
| 3 | 3661 | Attentive | Male | Mixed | 6 |
| 4 | 3067 | Attentive | Male | Tibetan Terrier | 7 |
| 5 | 3106 | Inattentive | Female | Mixed | 2 |
| 6 | 3418 | Inattentive | Male | Mixed | 2 |
| 7 | 3040 | Inattentive | Female | Labrador Retriever | 4 |
| 8 | 3043 | Attentive |  |  |  |
| 9 | 3241 | Attentive | Female | Golden Retriever | 4 |
| 10 | 3634 | Attentive | Female | Australian Labradoodle | 4 |
| 11 | 3121 | Inattentive | Male | Mixed | 10 |
| 12 | 3379 | Inattentive | Male | Wheaten Terrier | 1 |
| 13 | 3490 | Inattentive | Male | Mixed | 1 |
| 14 | 3700 | Attentive | Male | Mixed | 0 |
| 15 | 3451 | Inattentive |  |  |  |
| 16 | 3613 | Attentive | Female | Toy Poodle | 8 |
| 17 | 3259 | Attentive | Male | Pembroke Welsh Corgi | 0 |
| 18 | 3148 | Inattentive | Female | Mixed | 10 |
| 19 | 3607 | Inattentive | Female | Mixed | 3 |
| 20 | 3337 | Attentive | Female | Mixed | 4 |
| 21 | 3676 | Attentive | Male | Mixed | 3 |
| 22 | 3682 | Attentive |  |  |  |
| 23 | 3499 | Inattentive | Male | Maltese/Poodle Mix | 11 |
| 24 | 3166 | Attentive | Male | Labrador Retriever | 2 |
| 25 | 3298 | Inattentive | Male | Belgian Tervuren | 2 |
| 26 | 3811 | Inattentive | Female | Australian Shepherd | 0 |
| 27 | 3625 | Attentive | Male | Mixed | 3 |
| 28 | 3820 | Inattentive | Female | Mixed | 3 |
| 29 | 3805 | Attentive | Female | Mixed | 2 |
| 30 | 3832 | Attentive | Female | Mixed | 3 |
| 31 | 3745 | Inattentive | Female | German Shepherd | 8 |
| 32 | 3838 | Inattentive | Female | Mixed | 5 |
| 33 | 3853 | Inattentive | Male | Mixed | 9 |
| 34 | 3646 | Attentive | Female | Rat Terrier | 21 |
| 35 | 3751 | Inattentive | Male | Mixed | 2 |
| 36 | 3904 | Attentive | Male | Border Collie | 11 |
| 37 | 3880 | Inattentive | Male | Mixed | 8 |
| 38 | 3958 | Attentive |  |  |  |
| 39 | 3052 | Inattentive | Female | Labrador Retriever | 5 |
| 40 | 4006 | Attentive |  |  |  |

Table 2. Demographic data for Looking back study. Five dogs have missing data because the guardians declined to provide it.

Instruction sheet sent to guardians for Study 1


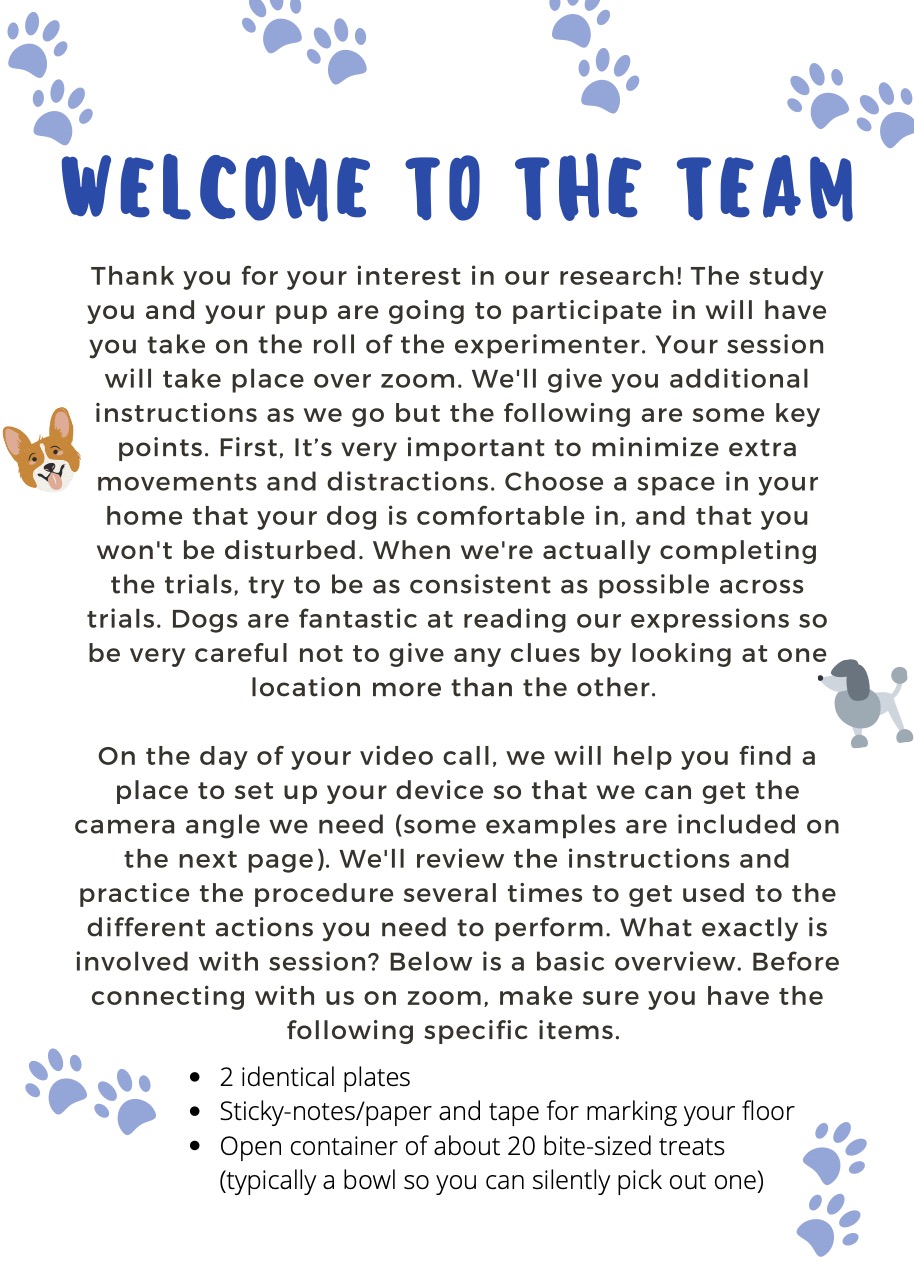


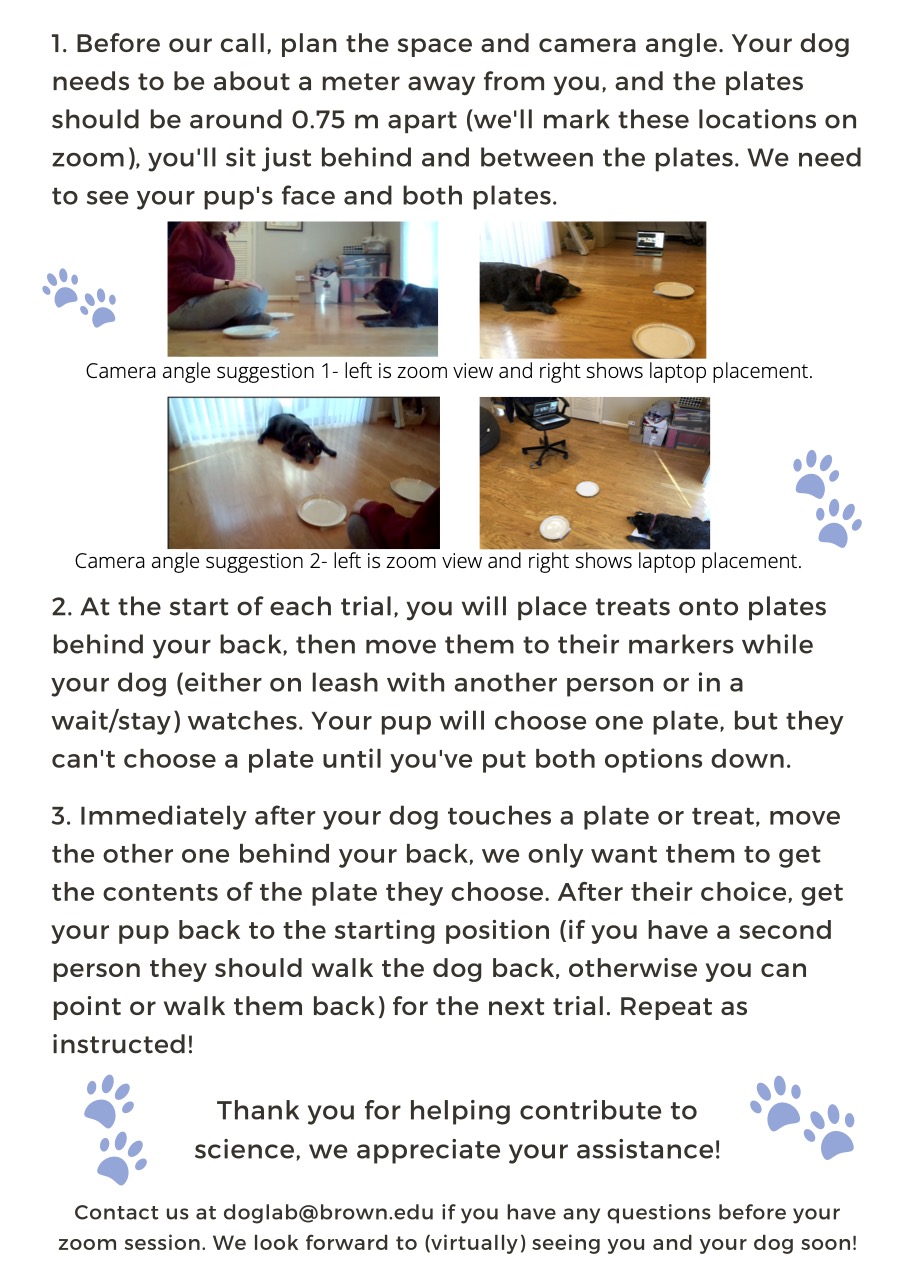


Script used for Study 1 by video experimenter:

*(Greet, standard intro chit chat*) First, I want to give you an overview of the structure of today’s session. We’ll go over a brief checklist of the items you need, we’ll get the room and camera angle set up, I’ll go over a series of instructions with you, then we’ll do some warm-up trials and finally our test trials. Throughout our zoom session today, I’m going to rely on you to make sure that [DOGNAME] is having a nice time. The main goal is that [DOGNAME] enjoys themselves, so if you think they need a break or want to stop early please just let me know! Normally I would be able to check on this myself but it’s difficult over zoom.

Okay – can you confirm that you have all the items ready? You need 2 plates/bowls, some tape and paper/sticky-notes, an open container of bite-sized treats you can easily grab. *(Confirm all the items are present and appropriate).* Finally, can you confirm you’re in a good open space in your home where there’s not likely to be interruptions? *(Confirm and move if needed).*

Great! We are going to make a T-shape on your floor. You want [DOG NAME] to be at least an arms-length away from you, aiming for one meter, so that you are on either long-end of the T. It’s most important that your dog starts in the same place before each trial.

Some people like to use natural borders in their home (i.e., start your pup right on the edge of your carpet) or you can put a sticky note or piece of tape on the ground where your dog will start, whichever works better. Please mark the spot now. *(wait for them to mark the spot).* Now that you know where you and [DOG NAME] will be, have a seat on the ground. We need to put down markers for where you’re going to set down your two plates. You want both plates to be diagonal off the edge of your knees where you can still easily reach them, but far enough away so that your pup can’t switch their choice too easily. *(provide feedback until they place the plates correctly)* Put a marker of some kind on the ground right underneath each of the plates. For each two-plate trial, you’ll move the plates from behind your back to these locations. For our warm-up trials you’ll put the plate between.

Now that we have all the markers placed, let’s move your device around so we can find the best camera angle. I need to be able to see both plate markers, as well as your pup’s face. In the instruction sheet we gave you there were some examples of placement, like on something high over your shoulder, or if your space is wide enough on the floor off to the side between your dog and the plate markers. You can also consider placing me around your dog’s height (like on a chair) off to the side between your dog and the plate markers.

*(WORK WITH DOG GUARDIAN TO FIGURE OUT THIS PLACEMENT)*

Now that we have the places marked and the camera set up, we can get started with the instructions for the practice trials. We’re going to start with using only one plate at a time so that both you and your dog can get a hang of what the rest of the trials will look like. First, I’m going to go over instructions for these practice trials, and then we’ll get started. If at any point you lose connection with me or my audio is skipping, just pause where you are and wait until we get re-connected. Sound good? *[GET GUARDIAN CONFIRMATION]*

***[IF ONLY ONE PERSON, PROCEED TO PAGE 4***]

***[IF A SECOND PERSON, ADDRESS DIRECTLY***]: Who wants to handle [DOG NAME]? [HANDLER NAME], your job is to situate [DOG NAME] about a meter away from [GUARDIAN/EXP NAME] at the start marker we just created. It’s important that you make sure [DOG NAME] is right in the middle and not shifted to the left or right. You’ll hold [DOG NAME] while looking down at the ground, trying to avoid looking at what [GUARDIAN/EXP NAME] is doing. Once [GUARDIAN/EXP NAME] says “OK” or whatever your pup’s release word is, you can loosen the tension in the leash and let [DOG NAME] move forward. As soon as they’ve touched the plate, call them back and get them set up to repeat the process. [HANDLER NAME], do you have any questions?

[GUARDIAN/EXP NAME], when I say it’s time to start, have a seat at your pre-determined spot and put one treat in the middle of one of one of the plates out of view of your dog (I find behind your back usually works best). Once the treat is in the center of the plate and your dog is ready to go, make sure your dog is watching you and put the plate down directly in front of you, between the two plate markers. Once you’ve set down the plate, put your hands on your knees and say your dog’s release word. As soon as [DOG NAME] touches the plate and eats their treat, return the plate behind your back

Don’t worry - i’ll keep repeating the instructions as we go!

Any questions? Okay let’s get started.

***[IF THEY CONSENTED TO VIDEO]*** On the consent form you were previously emailed you indicated that it was okay for me to record today’s session. I want to confirm that I have your permission to begin recording the session now? *[GET GUARDIAN CONFIRMATION]*

[HANDLER NAME] please get [DOG NAME] set up at the start marker. *(wait for dog to get set and provide remedial feedback if needed)*

[GUARDIAN/EXP NAME] please take one treat from your container and put it on the plate behind your back. Verify [DOG NAME] is watching and place the plate directly between your two markers. Return your hands to your knees, look down, and say your dog’s release word. *(watch dog guardian do this and make mental notes of any feedback)*

*(once choice has been made)* Great - [GUARDIAN/EXP NAME] please put the plate back behind your back and [HANDLER NAME] get [DOG NAME] reset.

*(give any feedback that’s required in the format “For this next trial…..”)* Okay second trial, [GUARDIAN/EXP NAME] please do that again - put one treat, verify [DOG NAME] is watching, place the plate in the middle. Return your hands to your knees and say your dog’s release word. *(watch dog guardian do this and make mental notes of any feedback)*

*(once choice has been made)* Great - [GUARDIAN/EXP NAME] please put the plate back behind your back and [HANDLER NAME] get [DOG NAME] reset.

*(give any feedback that’s required in the format “For this next trial…..” Assuming all is going well this should be the last trial)* Okay third trial, [GUARDIAN/EXP NAME] go ahead.

*(once choice has been made)* Great - [GUARDIAN/EXP NAME] please put the plate back behind your back and [HANDLER NAME] get [DOG NAME] reset.

*(repeat the above until dog and guardians are comfortable)* We’re going to move on to our two plate trials now!

[HANDLER NAME] your job is exactly the same, it’s just extra important that you don’t let [DOG NAME] go before [GUARDIAN/EXP NAME] says the release word. [GUARDIAN/EXP NAME] you’re now going to put out both plates onto your sticky notes in the exact same way you’ve been doing. I’ll tell you at the start which plate gets the treat (left or right), and which plate goes out first (empty or treat). For these trials, it’s also very important that you take away the unchosen plate immediately as we don’t want [DOG NAME] checking out both plates, only the one [he/she] picks. We define choice here as physically touching the plate, so if [DOGNAME] just approaches or looks over that doesn’t count. This means that if [DOGNAME] touches the empty plate, you have to immediately take away the plate with the treat on it, and they’ll get another chance to get a treat on the next trial. We’re going to repeat this process for 12 trials.

The phrasing of the cue can be a bit confusing so let me give you an example. For each trial, I will say, as an example, this is trial number 11, the treat goes on the right plate and the empty plate goes out first. You would then put the left plate out first with nothing on it, and the right plate with the treat on it out second.  Do you have any questions?

Great let’s get started. This is trial number [1-12], [HANDLER NAME] get [DOG NAME] set. [GUARDIAN/EXP NAME] the treat goes on the [LEFT/RIGHT] plate and the [TREAT/EMPTY] plate goes out first. *(once choice has been made)* Great - [GUARDIAN/EXP NAME] please put take away both plates and put them behind your back and [HANDLER NAME] get [DOG NAME] reset.  *(repeat the above for 12 trials)*

That’s it, we are all done – thanks for participating you all did a great job! We really appreciate you helping us out with our research! Can I answer any question for you before we sign off?

Script used for Study 2 by video experimenter:

1. Ask them if it’s ok to start recording
2. Start recording by hitting the record button on zoom
3. Remember to pin their video!
4. Introduce yourself
   1. “I am _______, thanks for participating today! Are you ok with this being recorded? This video is going to be on our password protected servers, no one except us will see it unless you explicitly give us permission.
5. Intro to experiment
   1. This is an experiment about how dogs interact with their people.
   2. We want to observe how dogs attempt to get treats from under the couch. This will then lead us to discovering what strategies dogs use to try to get the treats.
   3. Make sure to answer any questions they have about any of these concepts, you can talk about this section for as long as they want
6. Outline
   1. “I’m going to explain the process first, so you can ask any questions before you try”
7. Consent
   1. “You are going to put treats under the couch and then act in a specific way which I will explain in a second, then we are going to see whether your dog changes his strategy based on the way you act"
   2. “Does that make sense to you? Do you have any questions?
   3. Answer questions
   4. **Ask them to read statement**: “Today is _______. My name is ________. I understand the purpose and procedure titled “Looking Back” , and I agree to participate. I understand that either I or my dog may stop participation at any time. Any other people who appear in the background of this recording have agreed to be recorded, and if they are under the age of 18 I am their legal guardian and give permission for recording. [Optional: I also give permission for my participation to be used for later presentations of this work.]"
8. First I am going to explain fully what you will be doing
9. **Warm ups:**
10. "first we are going to make sure that your dog knows that there are treats under the couch - this is to set up our experiment. So you can now get your bag of treats and place it under the couch (or whatever place you have chosen). Make sure your dog is paying attention to this. And make sure they are in an unreachable spot.
11. There will be three trials in which you will grab a treat from the bag. Place one treat on the floor to the right of your dog, then one on the left of your dog, and lastly, back to the right.
12. Make sure you are also holding the reading object while doing these trials.
13. **Still Phase:**
    1. Now you will just just sit watching your dog for 30 seconds (I will turn on my stopwatch). Keep your hands in your lap, and watch your dog passively. I will let you know when you can stop.
    2. You can then embrace your dog and give them a treat!
    3. Now you will turn sideways (facing the camera), look down, and pretend to read the object in your hands for 30 seconds (I will turn on my stopwatch). Remember to NOT look at your dog for any reason. You are passively reading, and after 30 seconds, I will tell you to stop.
    4. You can then embrace your dog and give them a treat!
14. **Set up**
    1. **Assess camera angle**
       1. Look for a quiet corner of your house, where your dog might be less distracted, and try to get them to come over with you by calling them. Try not to use a treat or toy to lure them over
       2. More angled down than you think
          1. On the floor
          2. Also back up the camera more than you think
          3. Assess lighting: if too dark, ask them if they can turn on a light. If not, just keep going.
    2. Mention
       1. Things you will need:
          1. Camera
          2. Book, notebook, or readable piece of paper.
          3. Dog
          4. Ziplock bag full of dog treats
          5. Make sure that there is a location that your dog can not reach (e.g., under furniture such as a couch, armchair, bed, or dresser) are all good examples. Set up a camera (on your phone, computer, tablet or otherwise), to capture as much of the space as you can (it is helpful if you place your camera against a wall so the dog is not able to go behind the camera).
15. First you are going to play almost like a game with your dog that involves treats.
16. Then we are going to change things up.
17. You can stop at any time if you or your dog is uncomfortable

1. Reminders
   1. Try your best to keep your hands face up in your lap
   2. During the still phase, do not pet your dog, even if they put their head/paw on you
   3. Try to keep your face as neutral during the experiment - it is important to stay as still as possible for best data.
   4. Don’t give your dog any commands.
   5. Don’t worry if they walk away! Anything your dog does during the trial is totally fine. However, you can absolutely stop at any time if you or your dog is uncomfortable
   6. Ok, just to make sure, can you repeat to me what you will be doing
   7. Sounds great!
2. **Ready?**
3. **Begin**
   1. Time for thirty seconds, and when finished say “stop or all done”
4. Closing
   1. That was awesome! Thank you for your time, you and your dog did an amazing job!
   2. Do you have any other questions?
